# Habits in Pavlovian learning: Resistance to devaluation by sensory-specific satiety following extensive exposure to palatable tastes

**DOI:** 10.3758/s13423-025-02835-x

**Published:** 2026-02-17

**Authors:** Ana González, Isabel de Brugada

**Affiliations:** Mind, Brain, and Behavior Center (CIMCYC), Department of Experimental Psychology, University of Granada, Granada, Spain

**Keywords:** Pavlovian learning, Overtraining, Sensory-specific satiety, Habit, Tastes, Flavors

## Abstract

Most food preferences and aversions are acquired through experience. These learned behaviors arise from the positive or negative experiences that occur during (simultaneous taste–taste learning) or after (taste–consequence learning) consumption. Just as ingesting a food that causes nausea leads to a conditioned aversion, ingesting a highly hedonic or caloric food increases its preference. Appetitive preference learning has been extensively studied using the flavor preference conditioning paradigm. The latter involves presenting a preferred taste (i.e., sucrose; Unconditioned Stimulus [US]) with an initially neutral flavor cue (i.e., cinnamon; Conditioned Stimulus [CS+]), ultimately resulting in a conditioned preference for the CS+. Previous studies have shown how conditioned preferences are weakened after devaluing the US, suggesting that such preferences are based on flexible, goal-directed behavior guided by the activation of the US representation (Stimulus–Stimulus learning). The present study investigates whether the type of training (extensive or short) determines the learned content. Specifically, we explore whether initial learning involves flexible, goal-directed behavior that shifts to automatic, habitual behavior after extended training. Our findings reveal that while the minimally trained group showed a devaluation effect, the group given extended training continued responding even after the devaluation treatment. These findings are discussed in the context of modern obesogenic environments and in terms of their theoretical significance, given the limited evidence of habitual behavior in Pavlovian learning.

## Introduction

In their natural environments, animals must effectively select nutrient-rich foods while avoiding potentially toxic ones to ensure survival in resource-scarce settings. To accomplish this, animals develop food preferences that guide their dietary choices and optimize food intake. The development of flavor preferences has been extensively studied within the associative learning framework using procedures such as Conditioned Taste Aversion (where a stimulus is paired with gastric illness, e.g., Delamater et al., 2006) and Appetitive Conditioning (where a stimulus is paired with a palatable taste or nutrient, e.g., Harris et al., 2004). The latter, referred to here as *flavor preference conditioning*, has been widely studied and consistently demonstrated in animal models (e.g., Gil et al., 2021) and also investigated in human models (e.g., Gould et al., 2018).

The typical procedure for assessing flavor preference conditioning involves an initial phase in which a palatable taste, such as sucrose (Unconditioned Stimulus [US]), is paired with a neutral flavor cue, such as an almond aroma (Conditioned Stimulus [CS+]; e.g., Dwyer et al., 2009; Gil et al., 2021). Following this procedure, when animals are presented with the CS+ flavor alongside water or another previously experienced flavor that has not been paired with the US (CS−), a strong preference for the CS+ is observed (e.g., González et al., 2023). This conditioned preference has been proposed as a case of Pavlovian conditioning in which the representation of the neutral flavor cue becomes associated with either the sensory properties of the US (flavor–flavor learning, e.g., vanilla–sweet; e.g., Gil et al., 2021) and/or its post-ingestive consequences (flavor–nutrient learning, e.g., vanilla–calories; e.g., Harris et al., 2004). From this theoretical perspective, this preference relies on a Stimulus-Stimulus (S-S) association, in which the CS+ motivates consumption by activating the representation of the US (e.g., Dwyer, 2005). Evidence supporting this account comes from studies using US devaluation procedures. These studies have shown that acquired flavor preferences are weakened after devaluation treatments such as Conditioned Taste Aversion (e.g., Delamater, 2007; Delamater et al., 2006; Dwyer, 2005, Exp.1; Harris et al., 2004) or Sensory-Specific Satiety (Dwyer, 2005, Exp. 2; González et al., 2024, Exps. 1–2).

Devaluation procedures have traditionally been employed to determine whether a behavior is guided by an S-S association or automatically elicited by an environmental stimulus (S-R association; e.g., Adams, 1982; for reviews: Bouton, 2024; Watson et al., 2022). In S-S associations, a connection is formed between the representations of the CS and the US. As mentioned earlier, the conditioned response occurs when the presence of the CS+ activates the representation of the US in memory. In this scenario, if the reinforcer (US) is no longer valuable, behavior is updated, leading to a cessation or reduction of the response, indicative of goal-directed behavior. In contrast, S-R associations involve a direct connection with a response driven by the US. In this case, conditioned responding is stimulus driven, meaning it is automatically elicited. Therefore, as the representation of the US is not being processed, this behavior is immune to devaluation procedures. Thus, if behavior persists even after the reinforcer has been devalued, then this would indicate automatic behavior.

The distinction between flexible, goal-directed (S-S learning) and rigid, habitual behavior (S-R learning) has largely been studied in the context of instrumental learning (e.g., Thrailkill & Bouton, 2015). In contrast, within the Pavlovian learning paradigm, the study of S-R associations received little attention following the emergence of cognitive models of associative learning (Rescorla, 1988). In instrumental learning, it is widely believed that S-R associations—or habit learning—arise through repeated behavior (but see Bouton, 2024, for alternative proposals). Thus, while short training regimes tend to produce flexible and goal-directed behavior, extended training regimes lead to rigid, stimulus-driven behavior (e.g., Adams, 1982; Thrailkill & Bouton, 2015). In Pavlovian learning, however, few studies have attempted to demonstrate a similar dissociation with paradigms other than conditioned preference learning. Notably, such efforts have found no evidence of habitual (S-R) behavior: conditioned responding is consistently reduced following a US devaluation treatment (e.g., Holland, 1990, 2005; Holland et al., 2008).

A critical aspect of the abovementioned experiments on devaluation and flavor preference conditioning is that they were all conducted using short training regimes in which rats were exposed to the CS−US compound for a restricted period (e.g., Delamater et al., 2006; Dwyer, 2005; González et al., 2024, Exp. 1). To the best of our knowledge, the impact of extended training on the expression of the devaluation effect in flavor preference conditioning has only been tested once in rats (González et al., 2024). In this series of experiments, the authors trained rats to associate a neutral flavor (CS+) with a 10% sucrose solution (US), while another neutral flavor was presented in isolation (CS−). Afterward, the rats underwent a US-devaluation procedure using a Sensory-Specific Satiety procedure. This procedure involved presenting the animals with sucrose (devaluation) or water (control) on two counterbalanced days, after which their preference for the CS+ over the CS− was assessed. The results revealed that rats trained under a short-conditioning regime and pre-fed with sucrose expressed no preference for the CS+ compared with the water condition, showing a typical devaluation effect (Experiments 1–2). In contrast, rats trained under an extended-conditioning regime did not modulate their preference following the pre-feeding manipulation, instead exhibiting automatic behavior (Experiment 3). Despite the novelty of these findings, two potential limitations must be acknowledged. First, the effects of training were assessed by comparing different experiments that manipulated training type, rather than within a single experiment comparing extended and short training across groups. Second, the study did not fully demonstrate the selective effects of devaluation by using water as a control substance for pre-feeding. In Sensory-Specific Satiety procedures, a different US is typically included as a counterbalance to confirm that decreases in conditioned responding reflect a specific devaluation of the target US, rather than nonspecific changes such as shifts in motivational state. Without this control, reductions in responding during the test could simply reflect motivational effects of sucrose calories (e.g., satiety), which would be expected to decrease responding not only to the CS+ paired with sucrose but also to other CS+ associated with USs of comparable motivational value, even if their sensory properties differ, such as maltodextrin.

The present research aims to replicate the findings of González et al. (2024) using a more robust experimental design. To address the limitations of previous studies, we adopted a between-subject design in which one group was subjected to a short training procedure while another group experienced an extended procedure. The training consisted of pairing two CSs+ flavors with two different USs (sucrose or maltodextrin) and presenting another CS− alone. Both training groups had unrestricted access to the target substances for 6 hours per daily session, differing only in the number of training days: the short-training group completed half as many sessions as the extended-training group. It should be noted that even this “short” training would be considered “extended” relative to standard training procedures in preference conditioning. In typical flavor preference conditioning protocols, animals are usually given restricted amounts of solutions for a short period and across only a few training sessions (e.g., Gil et al., 2021) whereas in this procedure animals have ad libitum access to the target substances for many hours, even if only for a few days. However, following González et al. (2024, Experiment 2), rats trained under these conditions still showed a reduction in conditioned preference following the US devaluation procedure. Therefore, this training regime was designated as the ‘short’ condition, as it enabled a comparison of the same type of massive training over different total durations (3 vs. 6 days). The devaluation procedure was conducted using Sensory-Specific Satiety, where either sucrose or maltodextrin was presented before a choice test between CS+_SUC_ and CS+_MAL._ A devaluation effect would be evidenced by reduced consumption of a CS+ when its associated taste had been pre-fed. Conversely, the absence of a devaluation effect would be indicated if the rats consumed similar amounts of both CSs+ flavors after pre-feeding. It should be noted that throughout this article, the term ‘devaluation’ refers exclusively to the selective devaluation of the US.

## Methods

### Subjects and apparatus

The experiment used 32 non-naïve Wistar male and 24 naïve female rats (supplied by Janvier Labs and the Scientific Instrumentation Center of the University of Granada). Female rats weighed an average of 262 g (range: 221–308 g), while male rats weighed an average of 500 g (range: 431–570 g). The non-naïve rats had previously participated in a perceptual-learning experiment and were familiar with water deprivation, one-bottle testing, lithium chloride injections, and exposure to salt, hazelnut, and raspberry odors. None of these stimuli were used in the present experiment. All rats were randomly assigned to two groups of equivalent weight (Extended: N=28; *M*: 396.5 g/Short: N=28, *M*: 399.6 g) and baseline consumption. Each group had an equal number of males and females. The experiment was conducted in two separate batches, with one batch consisting of male rats and the other of female rats.

Rats were individually housed in translucent plastic cages (35 × 12 × 22 cm) with wood shavings as bedding. Due to their isolation, the rats were provided with environmental enrichment to mitigate any potential stressful effects resulting from this condition. The animals were maintained on 12-hour light/dark cycle throughout the entire procedure, with the light cycle initiating at 8:00 am. Throughout the experiment, the rats had unlimited access to chow and remained in their home cages during all phases of the procedure.

The experimental solutions were prepared daily with osmosis water and were presented to the animals in different types of containers depending on the phase of the experiment. During training, the solutions were delivered in bottles with metal stoppers, while during pre-feeding and two-tube tests, they were provided in centrifuge tubes with stainless steel, ball-bearing-tipped spouts. Bottles with metal spouts were used during training to prevent the rats from chewing the stoppers during the six-hour consumption period. Although bottles provide less precision in measuring intake than centrifuge tubes, they minimize stopper damage and prevent data loss during this phase. Consumption was measured by weighing the tubes before and after each procedure. The flavored solutions consisted of 0.05% Vanilla or Almond aroma (CS+) (Manuel Riesgo) combined with either 10% domestic sucrose or maltodextrin (2% monosaccharides, 7% disaccharides, 91% higher polysaccharides; Guinama) (US), both diluted in water or 0.05% caramel aroma (CS−) (Manuel Riesgo) diluted with water. Half of the rats received the vanilla aroma paired with sucrose, and the almond aroma paired with maltodextrin, while the other half received the opposite pairing.

The Ethics Committee for Animal Research at the University of Granada and the Junta de Andalucía (09/07/2024/097) approved all the procedures described in this article. These procedures were classified as low severity according to European guidelines. Animals were monitored daily by those responsible for animal welfare in the research center.

### Procedure

The general procedure was adapted from Experiments 2 and 3 of González et al. (2024), which based its Sensory-Specific Satiety treatment on that used by Dwyer (2005) (See Table 1). Animals were water-deprived beginning at 4:00 pm on the day before the experiment started. On Day 1, the rats received a water baseline consumption session, during which they had free access to water for 6 hours (9.30 am – 3.30 pm). This baseline procedure was conducted to habituate rats to the training session schedule and to assess any potential differences in overall consumption between groups. The same schedule was maintained throughout the training sessions.

**Table 1:** Overview of experimental procedure

Group	Training	Two-tube training	Preference test	Pre-feeding cycles
	*18 or 9 days*	*1 day*	*2 days*	*2 days*
*Ext.*	6×			
	(CS+SUC / CS+MAL / CS−)	W vs W	CS+_SUC_ vs CS- / CS+_MAL_ vs CS-	SUC / MAL ➔ CS+_SUC_ vs CS+_MAL_
*Short*	3×			

*Note.* “Ext” denotes the extended group. “CS+” denotes a conditioned stimulus, an aroma paired with an unconditioned stimulus. “CS−”refers to a conditioned stimulus not paired with the unconditioned stimulus (in this case, caramel). “SUC” represents sucrose, “MAL” refers to maltodextrin, “W” stands for water, “/” indicates counterbalanced days, while “6×” and “3×” represent the number of exposures each group received to each training compound

On Day 2, rats in the extended group began the training procedure. This consisted of a daily 6-hour exposure to one of the following solutions: CS+US_SUC_, CS+US_MAL,_ or the CS−. Access to the solutions was ad libitum. Over a total of 18 training days, rats in the extended group received six exposures to each solution (CS+US_SUC_, CS+US_MAL,_ and CS−). The order in which the solutions were presented was counterbalanced across days. Meanwhile, rats in the short group received 6-hour sessions of free water consumption until Day 10, when they began the same training procedure as the extended group until Day 18. Thus, the rats in the short group received half as many training days as the extended group (9 days, with three exposures to each solution). At the end of each 6-hour training session, all rats received 15 minutes of ad libitum access to water to ensure hydration during this intensive training phase. Continuous water access was not permitted in order to encourage consumption of the CS− solutions.

Following the completion of training, the schedule of the experimental sessions was modified to include two daily sessions providing restricted access to fluids (9.30 a.m. and 3.30 p.m.). This adjustment aimed to increase the animals’ level of thirst and ensure consumption during the test phases. Morning sessions were reserved for the experimental procedures involving the target solutions, while the afternoon sessions were used to rehydrate the animals through a 30-minute ad libitum water session. (twice as long as during training due to the greater fluid deprivation in this stage). On Day 20, rats received a two-tube water training session, during which they were presented with two tubes filled with water. This session lasted 20 minutes, and after 10 minutes, the experimenter interrupted the session, removed the tubes, and returned them to the same positions. This procedure was designed to familiarize animals with the tubes, the testing protocol and assess potential left/right side preferences.

On Days 21 and 22, a test was conducted to ensure the rats had acquired a conditioned preference following training. On this test, the rats were presented with CS+_SUC_ vs CS− or CS+_MAL_ vs CS−, with the order of presentation of each CS+ solution counterbalanced across rats. This preference test procedure was the same as the two-tube water session conducted during training, with one exception: after the first 10 minutes, the position of the tubes was changed, moving the tube on the left to the right and vice versa. Additionally, the initial position of the CS+ or the CS− (left/right) was counterbalanced across rats. All these procedures were implemented to ensure that any observed preferences were due to conditioning rather than confounding factors such as left/right position biases.

After the initial preference tests, the pre-feeding cycles began for 2 days (Days 23–24). Each pre-feeding cycle consisted of a pre-feeding phase and a preference test phase. During the pre-feeding phase, rats were presented with a 10 ml sucrose or maltodextrin solution for 20 min. After this phase, the animals rested for another 20 min before proceeding to the preference test phase. The final preference test was conducted in the same manner as the initial preference tests, except that the rats were presented with the two CSs+: CS+_SUC_ vs CS+_MAL_. This test was designed to selectively measure preference for the devalued versus the non-devalued CS+. For half of the rats, CS+_SUC_ was the devalued stimulus, while CS+_MAL_ served as the non-devalued stimulus. For the remaining rats, this arrangement was reversed, with sucrose devalued on the first day and maltodextrin on the second. An effect of specific devaluation would be indicated if the rats consumed less of the CS+ associated with the pre-fed solution compared with the other CS+. Conversely, a lack of difference in consumption between the two CSs would be interpreted as an absence of devaluation. An important consideration is that sucrose and maltodextrin are isocaloric. Therefore, any effects observed after pre-feeding are likely attributable to the devaluation of sensory properties rather than differences in caloric content. Notably, some authors have suggested that, despite their similar energetic value, these substances may differentially influence learning (e.g., Bonacchi et al., 2008; Elizalde & Sclafani, 1988; but see Dwyer, 2005). As discussed later, because the animals were trained under non–food-deprived conditions, their learning was likely driven more by the sensory qualities of the USs than by their motivational or caloric properties.

### Data analysis

Data were analyzed using General linear model null hypothesis testing, adopting a rejection level of *p* <.05. For mixed factorial analyses of variance, Greenhouse–Geisser corrections were applied when sphericity assumptions were violated. Effect sizes were estimated using partial eta squared and Cohen’s *d* tests. When required, post hoc analyses were conducted with Holm’s correction to control for multiple comparisons. All analyses were conducted in JASP (JASP Team, 2020).

For the training analyses, the short and extended groups were treated separately. Consumption data for each CS+US compound (sucrose and maltodextrin) were averaged across sessions, this average CS+ consumption was compared with CS– consumption for each session. A repeated-measures ANOVA was conducted with CS (CS+US/CS−) and Session as within-subject factors and Sex as a between-subject factor. Initial preference testing was analyzed using a repeated-measures ANOVA with CS (+ or −) and Flavor (Sucrose-paired or Maltodextrin-paired) as within-subject factors and Group and Sex as between-subject factors. Data from the pre-feeding phase were analyzed using a repeated-measures ANOVA, with Flavor (Sucrose or Maltodextrin) as the within-subject factor and Group and Sex as between-subject factors. The preference test after devaluation was analyzed using a repeated-measures ANOVA, with Pre-fed flavor (Sucrose or Maltodextrin) and Devaluation (CS+devalued or CS+non-devalued) as within-subject factors and Group and Sex as the between-subject factors.

All analyses in this experiment were conducted using frequentist statistics. Because the preference tests played a central role in the design, we complemented these analyses with Bayesian statistics to evaluate whether non-significant differences provided evidence in support of the null hypothesis. Bayes factors (BF₀₁) were computed using the Jeffreys–Zellner–Siow (JZS) prior (Rouder et al., 2009), with each model compared against the null model in the case of Bayesian repeated-measures ANOVA. Following Rouder et al. (2009), BF₀₁ values greater than 3 were interpreted as evidence in favor of the null hypothesis, whereas values below 1/3 were interpreted as supporting the alternative hypothesis. JASP applies the principle of marginality, ensuring that models with interaction terms also include the corresponding lower-order effects (Wagenmakers et al., 2018). Given the transitivity of Bayes factors, BFs for interaction terms were obtained by dividing the BF of the model including the interaction by that of the model with only the lower-order terms (Morey & Rouder, 2011).

### Transparency and openness

This study has not been preregistered. However, data from all the experiments are publicly available in the APA repository on the Open Science Framework (OSF: https://osf.io/b82ts).

## Results

Training phase analysis revealed significant main effects of CS, *F*(1,26) = 94.07, *p* <.001, *η*_*p*_^2^ = 0.78, and Session, *F*(5,130) = 17.29, *p* <.001, *η*_*p*_^2^ = 0.39, whereas no significant differences were observed for Sex, *F*(1,26) = 1.59, *p* =.21, *η*_*p*_^2^ = 0.058. Regarding interactions, significant effects were observed for CS × Sex, *F*(1,26) = 4.33, *p* =.04, *η*_*p*_^2^ = 0.14 and CS × Session, *F*(3.19, 83.04) = 7.68, *p* <.001, *η*_*p*_^2^ = 0.22 (Greenhouse–Geisser correction was applied due to violations in the sphericity assumption). Finally, the interactions Session × Sex *F* < 1, and CS × Session × Sex *F*(3.19, 83.04) = 2.32, *p* =.07, *η*_*p*_^2^ = 0.08 (Greenhouse–Geisser correction applied) were not significant.

**Fig. 1:**
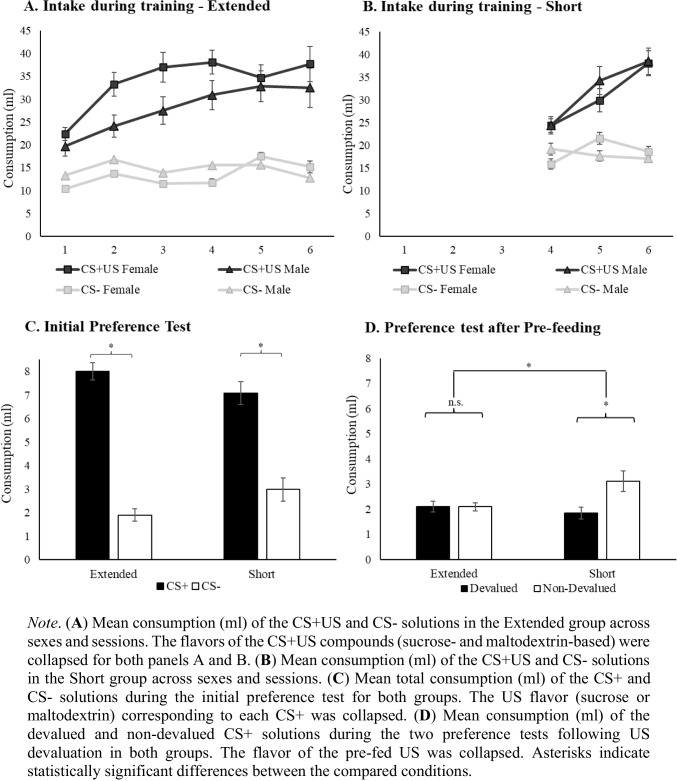
Group mean consumption during the training, initial preference testing, and devaluation test phases

Post hoc comparisons of the CS × Sex interaction revealed that both female, *t*(11) = 7.79, *p* <.001, *d* = 2.38, and male, *t*(15) = 5.81, *p* <.001, *d* = 1.54, rats consumed significantly more of the CS+US than the CS−. Consumption of the CS− did not differ between sexes, *t*(26) = 0.52,* p* =.15, *d* = 0.60. However, male rats consumed significantly less of the CS+US compared with females, *t*(26) = −2.34, *p* =.04, *d* = −0.69. A simple main effects analysis of the CS × Session interaction indicated that rats consumed significantly more of the CS+US than the CS− over the six sessions of conditioning, *F*(1,83.04) = 4,62, *p* =.04, *η*_*p*_^2^ = 0.05, for Session 1 and for the remaining 5 sessions, the lowest *F*(1,83.04) = 9.95, *p* =.004, *η*_*p*_^2^ = 0.10. Significant differences were also found in CS+US consumption over the six sessions of conditioning, *F*(5,83.04) = 24.43, *p* <.001, *η*_*p*_^2^ = 0.59, as well as for CS−consumption, *F*(5,83.049) = 2.45 *p* =.03, *η*_*p*_^2^ = 0.12. Hom’s post hoc comparisons revealed that CS+US consumption differed across sessions, being the difference between Session 1 and 6, *t*(27) = *p* <.001 *d* = −1.57_._ In contrast, Holm´s post-hoc comparisons revealed no significant differences in CS− consumption across sessions: 1 vs 6, *t*(27) = −1.30, *p* = 1, *d* = −0.24. To summarize, these results indicate that CS+US consumption increased over the sessions. In contrast, CS− consumption did not show a systematic increase across training sessions (see Fig. 1A).

The same analysis was conducted for the short group revealing a significant main effect of CS, *F*(1,26) = 74.58, *p* <.001, *η*_*p*_^2^ = 0.74, and Session, *F*(2,52) = 26.67, *p* <.001, *η*_*p*_^2^ = 0.50. The factor Sex did not reach significance, *F* > 1*.*The interactions CS × Session, *F*(2,52) = 22.84,* p <*.001,* η*_*p*_^*2*^* =* 0.46, and CS × Session × Sex *F*(2,52) = 4.07 *p* =.02, *η*_*p*_^2^ = 0.13 were significant, while the interactions CS × Sex, Session × Sex were not *F*s < 1.

A simple main effects analysis of the CS × Session × Sex interaction revealed no sex differences in consumption of the CS+US and CS− solutions across sessions (*F*s < 1). Female rats consumed more of the CS+US solution than the CS− solution across all sessions with the lowest *F*(1,26) = 4.31, *p* =.04, *η*_*p*_^*2*^* =* 0.14.. In male rats, no significant differences were found between consumption of the CS+US and CS− solutions on Session 1, *F*(1,26) = 2.20, *p* =.15, *η*_*p*_^*2*^*=* 0.07; however, significant differences emerged on Session 2, *F*(1,26) = 22.71, *p* <.001, *η*_*p*_^*2*^* =* 0.46 and Session 3, *F*(1,26) = 37.91, *p* <.001, *η*_*p*_^*2*^* =* 0.59, indicating greater consumption of the CS+US solution than the CS− solution. Additionally, significant differences in CS+US consumption were observed across sessions in both female, *F*(2,52) = 20.88, *p* <.001 *η*_*p*_^*2*^= 0.44, and male *F*(2,52) = 30.76, *p* <.001 *η*_*p*_^*2*^ = 0.54, rats. A Holm’s post hoc analysis revealed differences between Sessions 1 and 3 for both females, *t*(11) = −6.38 *p* <.001 *d* = −1.88, and males, *t*(15) = −7.60, *p <*.001* d* = −1.88. For the CS−, no significant differences were observed across sessions (*F* < 1). These results indicate a similar pattern to that observed in the extended group (see Fig. 1B).

Initial preference testing analysis revealed significant main effects of CS, *F*(1,52) = 108.26, *p* <.001, *η*_*p*_^2^ = 0.67, and Sex, *F*(1,52) = 39.31, *p* <.001, *η*_*p*_^2^ = 0.43 (female rats consumed less than male rats: Males: CS+ *M* = 8.58, *SE* = 0.41, CS− *M* = 2.76, *SE* = 0.44/Females: CS+ *M =* 6.15, *S*E = 0.26, CS− *M* = 2.00, *SE* = 0.30). The main effects of Flavor *F*(1,52) = 2.03, *p* = 0.15, *η*_*p*_^2^ = 0.03 (BF_01_ = 4.60) or Group *F* < 1 (BF_01_ = 5.35) were not significant. Additionally, a significant CS × Group interaction was observed, *F*(1,52) = 4.29, *p* =.04, *η*_*p*_^2^ = 0.07. All remaining interactions were nonsignificant: CS × Sex, *F*(1,52) = 3.06, *p* =.08, *η*_*p*_^2^ = 0.05 (BF_01_ = 1.04); CS × Flavor), *F*(1,52) = 2.66, *p* =.10, *η*_*p*_^2^ = 0.04 (BF_01_ = 0.63); Group × Sex, *F*(1,52) = 1.80, *p* =.18, *η*_*p*_^2^ = 0.03, (BF_01_=3); CS × Group × Sex (BF_01_ = 2.61); Flavor × Group (BF_01_ = 3.80), Flavor × Sex (BF_01_ = 5); Flavor × Group × Sex (BF_01_=2.75); CS × Flavor × Group (BF_01_ = 2.92); CS × Flavor × Group × Sex (BF_01_ = 1.48), *F*s < 1. Holm’s post hoc analysis of the CS × Group interaction revealed that consumption of the CS+ over the CS− differed in both groups, extended group: *t*(27) = 8.82 *p* <.001, *d* = 2.30; short group:* t*(27) = 5.89, *p* <.001, *d* = 1.53. Total consumption of the CS+ solution, *t*(54) = 1.77, *p* =.12,* d* = 0.37; BF_01_ = 1.41, and CS− solution, *t*(54) = −1.88 *p* =.12 *d* = 0.39; BF_01_ = 0.78, did not differ significantly between groups. These findings confirm that rats preferred the flavor paired with a palatable taste over the flavor that had been presented alone, demonstrating the effectiveness of the training procedure (see Fig. 1C).

The pre-feeding phase analysis revealed that the main effects of Flavor, *F*(1,52) = 1.87, *p =*.17, *η*_*p*_^2^ = 0.03, or Group, *F*(1,52) = 1.13,* p* =.29, *η*_*p*_^2^ = 0.02, were not significant (extended sucrose: *M* = 9.30, *SE* = 0.09; extended maltodextrin, *M* = 9.22, *SE* = 0.11/short sucrose, *M* = 9.24, *SE* = 0.1; short maltodextrin, *M* = 9.06, *SE* = 0.1). However, a significant main effect of Sex was observed, *F*(1,52) = 4.48, *p =*.04, *η*_*p*_^2^ = 0.07. Male rats consumed more during the pre-feeding phase than females (females: *M* = 9.08, *SE* = 0.09; males: *M* = 9.30, *SE* = 0.05). None of the interactions reached significance (*F*s* <* 1).

The preference tests after devaluation revealed a main effect of Flavor, *F*(1,52) = 11.61, *p* =.001, *η*_*p*_^2^ = 0.18, Devaluation, *F*(1,52) = 6.40, *p* =.01, *η*_*p*_^2^ = 0.11, and Sex, *F*(1,52) = 12.49, *p* <.001, *η*_*p*_^2^ = 0.19. No main effect of Group was observed, *F*(1,52) =1.81, *p*=.28, *η*_*p*_^2^ = 0.02 (BF_01_ = 2.33). The significant Flavor effect indicated that rats consumed more of both CSs+ solutions (devalued and non-devalued) when pre-fed with sucrose (total *M* = 5.09, *SE* = 0.34) compared with maltodextrin (total *M* = 4.07, *SE* = 0.35). Moreover, the significant effect of Sex showed that female rats consumed less overall on the test (devalued + non-devalued solutions, females: *M* = 3.44, *SE* = 0.32; males:* M* = 5.43, *SE* = 0.43). The interaction Devaluation × Group was significant, *F*(1,52) = 6.92, *p* =.01, *η*_*p*_^2^ = 0.11. Holm’s post hoc tests revealed that in the short group, consumption of the devalued CSs+ was significantly lower than non-devalued CSs+, *t*(27) *=* −3.65, *p* =.004, *d = −*0.69. However, in the extended group, there were no significant differences, *t*(27) = 0.07, *p* = 1, *d* = 0.01 (BF_01_ = 4.98)*.* Additionally, there were no significant differences between groups in consumption of the devalued, *t*(54) = 0.83, *p* = 1.00, *d* = 0.17 (BF₀₁ = 2.85) or non-devalued CSs+, *t*(54) = −2.51 *p* =.06 *d* = −0.53 (BF₀₁ = 0.42). Finally, consumption between devalued solutions in the extended group vs non-devalued solutions in the short group were nonsignificant: *t*(54) = −2.44, *p* =.06 *d* = −0.52 (BF₀₁ = 0.52), the same was true for consumption of devalued solutions in the short group vs non-devalued solutions in the extended group, *t*(54) = −0.76, *p* = 1, *d* = −0.16 (BF₀₁ = 2.70)The remaining interactions were not significant: Flavor × Group (BF_01_ = 4.10), Flavor × Sex (BF_01_ = 4.13), Devaluation × Group × Sex (BF_01_ = 2.1), Flavor × Devaluation × Sex (BF_01_ = 2.36), Flavor × devaluation × Group × Sex (BF_01_ = 2.07) *F*s < 1, Flavor × Devaluation, *F*(1,52) = 1.93, *p* =.17, *η*_*p*_^2^ = 0.03 (BF_01_= 1.05), Devaluation × Sex, *F*(1,52) = 2.45, *p* =.12, *η*_*p*_^2^ = 0.04 (BF_01_=1.78), Flavor × Devaluation × Group, *F*(1,52) = 1.18, *p* =.282, *η*_*p*_^2^ = 0.02 (BF_01_=1.94); Flavor × Group × Sex, *F*(1,52) = 1.97*, p* =.33, *η*_*p*_^2^ = 0.03 (BF_01 =_ 2.25), and Group × Sex, *F*(1,52) = 3.53,* p* =.06, *η*_*p*_^2^ = 0.06 (BF_01 =_ 0.90). Thus, the present results suggest that while the short group selectively reduced consumption of the devalued CSs+, rats in the extended group consumed both CSs+ regardless of US status, demonstrating insensitivity to specific devaluation (see Fig. 1D).

It is worth noting that the apparent lower consumption in the extended group, compared with the consumption of the non-devalued substances in the short group (although this difference does not reach significance), could suggest a case of nonspecific devaluation. This could imply an associative structure that is insensitive to specific devaluation but not necessarily a S-R one. However, this idea contrasts with previous results from González et al. (2024), which show an absence of a devaluation effect in the extended group when preference is assessed with a CS+ vs CS– comparison, as animals still exhibited an intact CS+ preference even when sucrose had been devalued.

## Discussion

These results demonstrate that the length of training in a flavor preference conditioning paradigm influences rats´ performance during a subsequent devaluation procedure. Rats trained with extended exposure to the CS+US compounds maintained a conditioned preference that was unaffected by US-specific devaluation. In contrast, rats trained with half the exposure to the CS+US compound selectively reduced consumption of the CS+ solution after pre-feeding with the corresponding US. This pattern of results suggests that, as in instrumental learning, conditioned preferences may shift over time from an initial flexible and goal-directed behavior (S-S association) to a much more rigid and automatic behavior (S-R association). Our findings, therefore, replicate those of González et al. (2024) while using a more robust experimental design, including tighter control over the selectivity of the devaluation procedure and conducting a direct comparison between the different training regimes.

González et al. (2024) used sucrose as the sole US, with water serving as the control for Sensory-Specific Satiety. In the present experiment, we employed two USs with similar caloric properties (sucrose and maltodextrin), which allowed counterbalancing for selective devaluation. Although some authors have suggested that the acquired preference for maltodextrin may be driven primarily by general motivational properties than by its specific sensory attributes (e.g., Bonacchi et al., 2008; Elizalde & Sclafani, 1988), Dwyer (2005) found that rats trained with low concentrations of sucrose and maltodextrin modulated their CS+ preferences according to the specific satiety procedure. This finding indicates that these preferences can indeed be mediated by sensory representations of the USs. In our experiment, US concentrations were relatively high (10%), but rats were not food-deprived during training, a condition that favors the formation of palatability-based, rather than nutrient-based, associations (Harris et al., 2000). The preference test was also conducted under non-deprivation conditions, in which flavor–flavor associations tend to be expressed even when calorie-based associations have also been acquired (e.g., González et al., 2023; Harris et al., 2000).

Another important consideration is that, as mentioned in the Introduction, although the Short group received less exposure to the substances than the extended group, overall exposure is still longer than that used in many flavor preference conditioning studies, which typically involve only 2–4 sessions of 10–30 min each (e.g., Dwyer, 2005; Gil et al., 2021). The decision to adopt this training protocol for the short condition was based on the study by González et al. (2024), which demonstrated that rats trained under both a long, standard protocol (Exp. 1) and a shorter, unrestrictive 6-hour protocol over three days (Exp. 2) still exhibited a CS+ devaluation effect. Taken together, these findings highlight that what animals learn depends not only on the number of training sessions but also on the type and duration of exposure given within each session.

As discussed in the introduction, the notion that extended practice leads to habit learning has been widely demonstrated in the instrumental paradigm but has historically been viewed as inconsistent with Pavlovian Learning (Bouton, 2021). Until now, only three studies have provided evidence that prolonged exposure to CS−US pairings results in a conditioned response that is resistant to devaluation procedures (González et al., 2024 with rodents; Mizunami, 2021 and Sato et al., 2021 with crickets). These studies represent notable exceptions in the Pavlovian learning literature. In this regard, Holland’s seminal experiments (e.g., Holland, 1990, 2005; Holland et al., 2008) demonstrated that, in a standard Pavlovian conditioning paradigm, the length of training did not affect sensitivity to US devaluation procedures.

It should be noted that Holland (2005) also investigated the effect of training length on the mediated learning of a food aversion, demonstrating that such learning occurs only after relatively brief training—but not prolonged training—of the CS−food relationship. Despite the inability of extensively trained CSs to produce aversion-mediated learning, these CSs clearly maintained associative access to a food representation, as evidenced by the observation that their food-approaching CRs remained sensitive to reinforcer devaluation. Thus, it seems reasonable to suggest that reinforcer devaluation and mediated learning involve different aspects of associatively activated stimulus representations, and access to these features changes over the course of training. However, in no case could one speak of a shift in behavioral control from S-S associations to S-R associations. Therefore, the present results, along with those of Mizunami (2021) and Sato et al. (2021), introduce a novel perspective, providing evidence of the potential to observe a shift in behavioral control within the context of Pavlovian conditioning.

On the other hand, it is important to note that although flavor preference conditioning has traditionally been interpreted as a form of Pavlovian conditioning, some researchers have proposed that, particularly in the context of conditioned taste aversion, flavor learning may also be understood within an instrumental learning framework (Dwyer et al., 2019; Li et al., 2013). According to this view, the flavored cue functions as a discriminative stimulus signaling the availability of an already preferred taste (the reinforcer), and consumption is considered the instrumental response. However, empirical evidence challenges a purely instrumental interpretation. For instance, flavor-nutrient preference conditioning has been shown to occur even when there is no instrumental contingency between the amount of flavored cue (CS+) consumed and the amount of nutrient received during training, such as when a fixed amount of nutrients is administered via intragastric infusions regardless of total CS+ consumption (e.g., Ackroff et al., 2012). Similarly, flavor–flavor learning has been demonstrated under conditions where animals do not voluntarily consume the solution, as in paradigms assessing orofacial responses (Forestell & LoLordo, 2003). Although these findings provide strong support for the involvement of Pavlovian mechanisms in flavor preference conditioning, it is important to note that instrumental contributions cannot be completely ruled out in the present design. The 1:1 relationship between CS+ consumption and US presentation allows CS+ intake to directly influence the amount of reinforcer received. Thus, this interpretation remains plausible. Nonetheless, as suggested in the context of conditioned taste aversion, it is plausible that both Pavlovian and instrumental processes operate in parallel during the acquisition of flavor preferences (Dwyer et al., 2019).

The present findings encourage further testing of other features of habitual behavior in flavor preference conditioning such as the degree of resistance to extinction or contextual dependence (Bouton, 2024). For example, with respect to extinction, one might hypothesize that preferences acquired through extended training—due to their reliance on S-R associations, which do not involve the activation of the US in memory—would be less affected by repeated presentation of the CS + in the absence of the US (Harris et al., 2004). In contrast, in a minimally trained group, repeated presentation of the CS+ without the US should degrade the S-S association, thereby weakening the conditioned response. Surprisingly, findings from the instrumental paradigm suggest that habitual responding is not necessarily protected from extinction (e.g., Dickinson et al., 1998; Thrailkill et al., 2018). One potential explanation for this apparent contradiction is that extinction testing may introduce a contextual change (Bouton, 2024). Specifically, during training, multiple CS−US pairings occur, establishing a stable CS−US association. However, the re-presentation of the CS+ in the absence of the reinforcer during extinction testing could be sufficient to create a shift in contextual conditions, thereby influencing the expression of the conditioned response. There is an ongoing debate regarding extinction in flavor preference conditioning. While several studies have demonstrated that this form of learning is highly resistant to extinction under standard training procedures (e.g., Albertella & Boakes, 2006; Harris et al., 2004), others have observed extinction effects when specific test conditions are met (Badolato et al., 2021; Delamater, 2007; for a review, see Hall, 2022). Although such effects have been observed using conventional flavor preference conditioning protocols, Delamater et al. (2021) recently found no difference in the pattern of extinction between standard and overtraining conditions. Whether overtraining, as applied in the present study, modifies extinction processes remains to be determined.

Another important factor in determining whether an instrumental response is habitual or goal-directed is contextual sensitivity: habits are typically context-dependent, whereas goal-directed behaviors may generalize across conditions different from those established during training (e.g., Bouton, 2021, 2024; Steinfeld & Bouton, 2021; Thrailkill & Bouton, 2015). A worthwhile next step would be to study whether sensitivity to context change differs after short versus extended training with this flavor preference learning paradigm. Interestingly, Sato et al. (2021) demonstrated that, contrary to what is observed in instrumental learning, Pavlovian habitual behavior was unaffected by contextual changes. A key difference between Instrumental and Pavlovian conditioning preparations that could account for these inconsistencies is the presence/absence of a discriminative stimulus during training. In instrumental learning, free-operant procedures are often employed, where no discrete stimulus is explicitly present during training. In such cases, the context itself is assumed to act as the stimulus (S) responsible for forming the habit (e.g., Thrailkill & Bouton, 2015). Conversely, in Pavlovian Learning, the discrete conditioned stimulus (CS+)—rather than the context—is associated with the response and drives the conditioned behavior. Given this distinction, it is plausible that introducing a discriminative stimulus during an extended operant learning procedure may reduce the contextual dependence of instrumental habits. With the presence of a stimulus acting as a reliable predictor of the outcome, the context would no longer serve as the primary cue for activating the operant response (Bouton, 2024).

Distinguishing between S-S/S-R learning has been the focus of both theoretical and applied research. Habitual behavior is widely regarded as a valuable form of learning in our daily lives, as it allows for the automatic execution of behaviors without the need for cognitive resources (Bouton, 2024; Watson et al., 2022). However, the rigid and automatic nature of habitual behaviors, which operate independently of their consequences, has also been studied as a model for maladaptive behaviors, including compulsions (e.g., Gillan et al., 2016) and substance abuse (e.g., Hogarth et al., 2013). Another maladaptive behavior associated with excessive habitual learning is overeating (e.g., Horstmann et al., 2015). In this regard, the process of acquiring conditioned preferences is closely linked to feeding behavior, playing a critical role in processes underlying food choices and portion size estimation (Berthoud et al., 2021). The results reported here suggest a possible mechanism by which modern societies could provide an ideal environment for automatic food consumption (González et al., 2024). Given the widespread exposure to highly caloric and palatable foods in today’s obesogenic environments, it is conceivable that S-R learning is set in motion, driving overconsumption. This automatic activation could even lead to consumption in inappropriate situations—such as when an individual is physiologically satiated—triggered solely by exposure to flavored cues.

As a limitation, it should be noted that no final test was conducted to assess the effectiveness of the US devaluation procedure. This is important because one could argue that the absence of a CS+ devaluation effect in the extended group might be due to sucrose becoming more resistant to losing its value after prolonged exposure. Consequently, this outcome would not necessarily imply a shift from an S-S to an S-R associative structure, as the CS+ could still activate the representation of a US that retains its value. An additional consideration concerns the use of a single, non-counterbalanced flavor (caramel) as the CS−, included to simplify the procedure. This stimulus served to demonstrate the basic preference effect (CS+ vs. CS−) and to determine whether preferences differed between CS+ solutions paired with different USs (maltodextrin or sucrose). While full counterbalancing would have been ideal, prior evidence suggests that the preference effect is highly robust; therefore, this limitation is unlikely to have influenced the main findings.

In conclusion, the present results contribute significantly to the literature on associative learning while also shedding light on a potential mechanism that may underlie excessive food intake in the context of the current obesity epidemic. Furthermore, this research firmly consolidates the initial results of González et al. (2024) with a more robust design and using a sample of male and female subjects, which also overcomes the sexual bias in the animal research literature, where male rats are predominantly used as experimental subjects. Finally, these insights could also inform the design of targeted prevention and treatment strategies for certain maladaptive behaviors.

## Data Availability

The data on which study conclusions are based are publicly available in the APA´s repository of the Open Science Framework (OSF): https://osf.io/b82ts
